# Evidence-based choices of physicians: a comparative analysis of physicians participating in Internet CME and non-participants

**DOI:** 10.1186/1472-6920-10-42

**Published:** 2010-06-10

**Authors:** Linda Casebeer, Jennifer Brown, Nancy Roepke, Cyndi Grimes, Blake Henson, Ryan Palmore, U Shanette Granstaff, Gregory D Salinas

**Affiliations:** 1 CE Outcomes, LLC, 107 Frankfurt Circle, Birmingham, AL, 35211, USA; 2 MedscapeCME, 370 Seventh Avenue, Suite 1101, New York, NY 10001-3967, USA

## Abstract

**Background:**

The amount of medical education offered through the Internet continues to increase, providing unprecedented access for physicians nationwide. However, the process of evaluating these activities is ongoing. This study is a continuation of an earlier report that found online continuing medical education (CME) to be highly effective in making evidence-based decisions.

**Methods:**

To determine the effectiveness of 114 Internet CME activities, case vignette-based surveys were administered to U.S.-practicing physicians immediately following participation, and to a representative control group of non-participants. Survey responses were analyzed based on evidence presented in the content of CME activities. An effect size for each activity was calculated using Cohen's *d *to determine the amount of difference between the two groups in the likelihood of making evidence-based clinical decisions.

**Results:**

In a sample of 17,142 U.S. physicians, of the more than 350,000 physicians who participated in 114 activities, the average effect size was 0.82. This indicates an increased likelihood of 48% that physicians participating in online activities were making clinical choices based on evidence.

**Conclusion:**

Physicians who participated in online CME activities continue to be more likely to make evidence-based clinical choices than non-participants in response to clinical case vignettes.

## Background

Continuing medical education (CME) activities provide opportunities for medical practitioners to keep up with new information affecting the delivery of medical care, and ongoing participation is required by most physician state licensing boards [1,2]. Participation in CME or other medical education activities is also required by the licensing boards for other types of healthcare providers, such as physician assistants and nurse practitioners. CME providers sponsor a variety of activities, such as courses, regularly scheduled series, or enduring materials, defined as instructional materials that can be accessed at a time chosen by the participant.

The number of hours of Internet-based enduring materials provided by Accreditation Council for CME (ACCME)-accredited providers increased dramatically in recent years, from 16,802 hours in 2002 to 57,944 hours in 2008 [3,4]. This three-fold increase was accompanied by an even larger increase in the number of participants choosing Internet-based enduring materials; the number of physician participants increased from 305,410 individuals in 2002 to 4,365,014, nearly a ten-fold increase.

Given the increasing number of CME activities offered on the Internet, and the even larger growth of participation in these activities, assessing the effectiveness of Internet-based CME is crucial. Reviews of studies comparing results from online and traditional CME materials conclude that Internet-based CME was as effective as the traditional CME delivery formats [5,6]. A recent review analyzed data pooled from published comparisons of participating in Internet-based CME activities vs. traditional CME activities and comparisons of participating in Internet-based CME activities vs. not participating in CME activities [7]. The review concluded that Internet-based CME improved participant knowledge, skills, and practice decisions, with results that were comparable to those obtained after participation in traditional CME activities.

The goal of this study was to assess the evidence-based decisions in response to clinical case vignettes by physicians participating in CME activities of varied formats and to compare those decisions with those of a similar group of physicians who did not participate in the CME activities. The CME activity formats included case-based, multimedia, and interactive text. We hypothesized that physicians who participated in each type of Internet CME activity would more frequently make evidence-based clinical choices in response to clinical case vignettes when compared to physicians who did not participate.

## Methods

To assess the evidence-based choices of physicians who participate in Internet CME activities, a group of 114 different Internet CME certified stand-alone activities posted during 2006-2008 was studied, including activities in the following formats: case-based, multimedia, and interactive text. 114 Internet CME activities were identified as eligible for assessment by meeting the following criteria: 1) designed for physicians, 2) posted between January 1, 2006 and December 31, 2008, 3) certified for CME credit (1 credit for majority of activities), and 4) presented in an on-demand archived format. Of the 114 Internet CME activities assessed during the period of the study, 40 were interactive CME case activities, 64 were interactive text-based activities, and 10 were multimedia activities. Interactive CME activities contain questions within the activity that participants respond to and receive immediate feedback, usually involving patient cases. Interactive text-based activities are mainly conference coverage, special reports, and basic clinical updates. Multimedia activities are mainly live or roundtable presentations with video lectures.

### Participant Selection

The controlled trial conducted compared the evidence-based clinical choices of a group of 8,550 participant physicians with those of a demographically matched control group of 8,592 non-participant physicians. Following participation, physicians were asked to respond to a series of clinical case questions related to application of the CME content to practice. Physicians who participated in these activities were eligible for inclusion in the study if they practiced in the U.S., represented the target audience for the activity, and completed assessment questions following participation. A random sample of participants meeting the eligibility criteria for each activity was drawn from each overall participant group. A demographically similar group of non-participant physicians selected at random from the American Medical Association (AMA) Master File was recruited to participate and also respond to the same clinical case questions. An average total sample size of between 100 and 200, with a minimum of 50 participants and non-participants, was used for individual activities. A sample size of 50 is the minimum number required for sufficient statistical power (p < 0.05). Participant and non-participant samples were matched on the following characteristics: physician specialty, degree, years in practice, whether or not direct patient care was their primary responsibility, and the average number of patients seen per week with the disease of interest. As Medscape members are likely to have participated in more than one activity, it is likely that individuals are recorded multiple times in the total participant number of 8,550. However, for each activity, only one score per individual is recorded.

### Assessment

A consistent assessment approach was developed and used across all of the 114 CME activities included in the study. This evaluation approach included: 1) using brief case descriptions to assess clinical practice choices, 2) presenting clinical choices using a multiple-choice format, 3) using a standard hypertext mark-up language programming approach to presenting assessment questions, 4) applying this assessment approach to specific content reflected in each individual activity, and 5) collecting assessment data from CME participants in each individual clinical assessment. Case vignette studies were reviewed by Western Institutional Review Board (WIRB; Olympia, WA) in 2004 prior to this study.

A standard assessment template consisting of two clinical vignettes and five to eight clinical questions using a multiple choice format was developed; evidence-based responses to the case vignettes were identified from content and references developed by the faculty for each activity. Content for the activities was written and referenced to clinical evidence by the faculty member for each activity. Only content referenced to peer-reviewed publications or guidelines was considered eligible for the development of clinical case questions. Case vignettes and the assessment questions were developed by clinical experts who were not involved in the design and content of the CME activity. Content validity of the case vignettes was established by review from physician medical editors of the online portal; editors represented the appropriate clinical area for each set of case vignettes. An example case and assessment questions are shown in the Appendix.

### Analysis

A statistical analysis software package (Statistical Package for Social Sciences 17.0; SPSS; Chicago, IL) was used in data extraction and transformation, and statistical analyses. Participant and non-participant case vignette responses were scored according to their concordance with the evidence-informed content presented within each activity. Overall mean scores and pooled standard deviations were calculated for both the participant and non-participant groups for each of the activities. These were used to calculate the educational effect size using Cohen's *d *formula (i.e., the difference in mean divided by the square root of the pooled standard deviation) in order to determine the average amount of difference between participants and non-participants [8]. Effect size representing the difference between the two groups was expressed as a percent of non-overlap between participants and non-participants.

## Results

### Participation

Over 350,000 physician participants were recorded for the 114 selected activities. A total of 17,142 physician responses to assessment questions in 114 activities were analyzed; of these, 8,550 were responses from participants and 8,592 were from the control group of non-participants. Demographics of participants are presented in Table 1. These physician characteristics were compared to the physician characteristics and distribution in the U.S. presented by the American Medical Association Masterfile [9] and did not significantly differ overall.

**Table 1:** Demographics of physician Internet CME participants and control group

Physician Characteristics	Participant Group N = 8550	Control Group N = 8592
Age, years		
Average	50.6	50.8
SD	10.7	9.2
Years since graduation from medical school		
Average	23	23.4
SD	11.1	9.4
Gender, number (%)		
Female	2,651 (31)	2,406 (28)
Male	5,899 (69)	6,186 (72)
Degree, number (%)		
DO	1,112 (13)	945 (11)
MD	7,438 (87)	7,647 (89)
Direct patient care as major professional activity, number (%)	7,866 (92)	8,076 (94)

### Evidence-Based Performance

Of the 114 Internet CME activities assessed for this study, 40 were interactive CME case activities, 64 were interactive text-based activities, and 10 were multimedia activities. Overall, the average effect size for the 114 Internet CME activities was 0.82, and the non-overlap percentile, representing the non-overlap between participants and non-participants in evidence- based responses, was 48%. Thus, as a whole, participants in the activities are 48% more likely than non-participants to provide their patients with evidence-based care, demonstrating a positive educational impact. The effect size by educational format is shown in Table 2. The effect by therapeutic areas is represented in Table 3. For this analysis, programs were categorized by therapeutic area for the following categories (activity topics are examples and not exclusive): cardiovascular disease (included activities focused on hypertension, dyslipidemia, heart failure, etc.), endocrinology (osteoporosis, diabetes, hypogonadism), gastroenterology (erosive esophagitis, ulcerative colitis), infectious disease (HIV/AIDS, influenza), pain management/neurology (multiple sclerosis, breakthrough pain, pain management), obstetrics/gynecology (contraception, hormone therapy), oncology (breast cancer, lung cancer), psychiatry (bipolar disorder, depression, autism), pulmonary disease (asthma, COPD), rheumatology (rheumatoid arthritis, ankylosing spondylitis), urology (overactive bladder, erectile dysfunction), and other (cultural competency, end of life care).

**Table 2:** Effect size of 114 Internet CME activities by format

CME Activity	N	Effect size (average)	% non-overlap between participants and non-participants
All Internet CME activities (n = 114)	17,142	0.82	48%

Interactive text-based (n = 64)	10,009	0.58	37%

Interactive case-based (n = 40)	5,320	1.08	58%

Multimedia (n = 10)	1,813	1.26	64%

**Table 3:** Effect size of 114 Internet CME activities by therapeutic area

Therapeutic area	# activities	Average effect size	% non-overlap between participants and non-participants
Cardiovascular Disease	18	0.72	44%

Endocrinology	5	0.52	34%

Gastroenterology	3	1.09	59%

Infectious Disease	13	0.66	41%

Pain Management/Neurology	11	0.73	44%

Obstetrics/Gynecology	8	0.98	55%

Oncology	8	0.96	54%

Psychiatry	24	1.06	58%

Pulmonary Disease	3	1.06	58%

Rheumatology	9	0.73	44%

Urology	10	0.59	38%

Other*	2	0.69	43%

*Other includes: Cultural competency, End of life care

## Discussion

From our work in analyzing a large sample of physician participants in 114 different Internet-based CME activities, combining the analyses presented herein with those presented in a previous report [10], it is clear that these Internet-based CME activities were effective; responses of CME participants to questions about the case vignettes were more likely to reflect evidence-based clinical choices than the responses of matched non-participants. These findings are consistent with the recent meta-analysis by Cook et al. concluding that Internet-based CME improved participant knowledge, skills, and practice decisions, with outcomes that were comparable to those obtained after participation in traditional CME activities [11]. The data also supports Wong's assertion, in a letter to the editor in response to Cook's meta-analysis - the data puts to rest the issue of whether this innovative educational method is efficacious [12].

Internet CME participants continue to demonstrate the usefulness of interactive Internet CME activities for experienced clinicians, as the participants in the study had an average of 20 years of experience in practice. The searchability of the Internet at the time that a clinician has a question may also contribute to the effectiveness of Internet CME activities [10].

Several recent reports develop consistent methods for comparing outcomes and applying the methods to multiple Internet-based CME activities. In one study, standardized tests administered before and after participation in courses offered at a Canadian CME web site revealed increases in participant knowledge, confidence, and self-reported change in practice patterns resulting from participation [13]. Improvements were noted for the majority of the 10 different courses that were assessed. In another recent study, standardized questionnaires including questions about case vignettes were administered to participants who had completed CME courses offered at a U.S. CME web site [10]. The results reported here support a recent comparison of the responses of CME-participants with the responses of matched non-participants that revealed that participants had a higher likelihood of making evidence-based clinical choices for 48 different courses [10].

The analysis reported here used responses to questions about clinical vignettes to measure the effectiveness of the CME activities, which is an indirect method of assessing a physician's practice patterns. However, clinical case vignettes have been shown to be valid tools for measuring the quality of clinical practice [14,15]. A limitation of the study is that the questions were administered immediately after participation in the CME activity; thus these analyses did not assess whether the improvements in physician performance were maintained over time. We also did not assess the effects of participating in the CME activities on patient health outcomes. Strengths of the study include the large number of physician participants and the varied Internet CME formats assessed.

Another possible limitation of these analyses is the exclusion of non-physician healthcare providers. The rapid growth in the number of non-physician participants in Internet-based CME activities parallels that of physician participants [4]. Furthermore, in 2008, more non-physician participants chose Internet-based enduring materials than any other ACCME-accredited CME category [4]. Given the predicted shortage of physicians and the increasing costs of health care, the roles of non-physician healthcare providers, such as nurse practitioners and physician assistants, are likely to continue to expand, and effective CME will be important in ensuring continued quality medical care [16].

## Conclusion

In summary, this study demonstrated that physicians who participated in varied formats of selected Internet CME activities were more likely, following participation, to make evidence-based clinical choices in response to case vignettes than non-participants. These data support the assertion that Internet CME activities are effective and offer a searchable, credible, available on-demand, high-impact source of CME for physicians.

## Competing interests

Authors are employees of CE Outcomes, LLC or MedscapeCME. They have no other potential conflicts of interest to declare.

## Authors' contributions

All authors have participated in the design of the study, the review of the data and the writing of the article. SG and BH were principally responsible for the data analysis. All authors read and approved the final manuscript.

## Appendix: Example of case vignette and assessment questions

Ms. Davis is a 45-year-old woman who presents to the primary care clinic with complaints of "stiffness." She notes that over the past year, she has noticed that she is slower than she has been previously, and it now takes her an hour to complete a 3-mile walk, when it used to take only 30 minutes. She also notes that she seems to "take a lot more steps" when she is walking. On examination, you note some mild bradykinesia and rigidity that is more notable in her left side. She has slowed finger and foot tapping speed on the left. Her writing is small. She has no tremor. Magnetic resonance imaging is performed which is unremarkable, and blood tests are all normal.

1. **What treatment options would you consider? **(select only one)

▪ Dopamine agonist

▪ Deep brain stimulation

▪ Physical therapy

▪ Amantadine

**Case #2 continued: **Ms. Davis is diagnosed with Parkinson's disease and started on the appropriate therapy. Her initial response to therapy is good. She has less bradykinesia and feels like she is walking well. She remains stable for 3 years. When you see her at her 4-year visit, you note increasing rigidity and slowness. She is started on levodopa, with a good clinical response for a few months, but she develops some "extra" movements where her arm "flings out" about an hour after her dose.

2. **What would you do at this point? **(select only one)

▪ Reconsider the diagnosis

▪ Increase her dose of levodopa

▪ Add another agent

▪ Refer to a neurologist

## Pre-publication history

The pre-publication history for this paper can be accessed here:

http://www.biomedcentral.com/1472-6920/10/42/prepub
